# *In vivo* CRISPR-mediated activation of cardiogenic genes to reprogram cardiac fibroblasts

**DOI:** 10.3389/fcell.2026.1812941

**Published:** 2026-04-22

**Authors:** Suchandrima Dutta, Sophie Chen, Waqas Ahmad, Wei Huang, Yigang Wang, Jialiang Liang

**Affiliations:** 1 Department of Pathology and Laboratory Medicine, College of Medicine, University of Cincinnati, Cincinnati, OH, United States; 2 Department of Internal Medicine, College of Medicine, University of Cincinnati, Cincinnati, OH, United States

**Keywords:** cardiac fibroblasts (CFs), cardiac regeneration, cardiomyocytes (CMs), cell reprogramming, CRISPR activation (CRISPRa), myocardial infarction, vascular smooth muscle cells (VMSCs)

## Abstract

**Background:**

We previously demonstrated that fibroblasts can be reprogrammed through a proliferative progenitor stage to drive both cardiomyogenesis and neovascularization. However, it remains to be determined whether cardiac fibroblasts (CFs), the primary mediators of post-injury remodeling, retain the plasticity to be concurrently redirected into cardiovascular lineages within the *in vivo* environment.

**Methods:**

This study aimed to activate endogenous cardiogenic genes (*Gata4*, *Nkx2*.5, *Tbx5*, *Isl1*, and *Smarcd3*, referred to as *GNTIS*) in CFs using a CRISPR activation (CRISPRa) system. CFs isolated from fibroblast-specific transgenic dCas9 mice were used to validate cardiogenic gene activation mediated by single-guide RNAs and VP64 activators delivered *via* adeno-associated viral vectors (AAVs). The reprogramming potential of CFs into cardiovascular lineages was further evaluated in dCas9 mice subjected to myocardial infarction followed by administration of pooled AAVs.

**Results:**

Individual *GNTIS* sgRNAs effectively upregulated their respective targets in the transfected CFs. The pooled sgRNA^*GNTIS*^ induced cardiac-like phenotypes in the CFs, as demonstrated by the Nkx2.5 cardiac progenitor reporter and increased cardiac differentiation markers. Subsequently, AAV-sgRNA^*GNTIS*^ was assessed *in vivo* in dCas9 mice following myocardial infarction. While global cardiac function did not reach statistical significance, *GNTIS* activation improved key hemodynamic parameters, effectively preserving cardiac performance compared to controls. Immunostaining further revealed that *GNTIS*-activated CFs exhibited the potential for transdifferentiation into cardiomyocyte-like or vascular smooth muscle cell-like lineages within the infarcted heart, which was not observed in the control group.

**Conclusion:**

This CRISPRa transgenic model provides a foundational proof of concept for *in vivo* reprogramming of CFs into cardiovascular cells.

## 1 Introduction

After myocardial infarction (MI), cardiomyocytes (CMs) undergo cell death and are replaced by fibrous scars composed of cardiac fibroblasts (CFs) and extracellular matrix (Travers et al., 2016). CFs, which possess a high capacity to reenter the cell cycle and exhibit phenotypic plasticity, become a major cell population in the heart after MI (Ma et al., 2017). CFs tend to differentiate into myofibroblasts for natural self-healing; however, excessive fibrosis leads to adverse remodeling and eventual heart failure (Weber et al., 2013). Unfortunately, there are no effective therapies to prevent or reverse cardiac fibrosis. Promisingly, CFs are an attractive target for *in vivo* reprogramming to replace fibrous tissue with newly induced CMs (iCMs) by overexpressing defined cardiogenic transcription factors (Qian et al., 2012; Song et al., 2012). The advantages of *in vivo* cell reprogramming include direct gene delivery in the heart, avoiding the need for surgical cell transplantation, thereby minimizing risks of graft-induced arrhythmia, immunogenicity, or tumorigenicity (Srivastava and DeWitt, 2016; Ahmad et al., 2025).

Given the multifaceted pathological changes that occur in MI, reprogrammed iCMs often require additional nutritional or extracellular matrix support from other cell types to effectively repair the damaged heart (Mathison et al., 2012; Yamakawa et al., 2015; Jin et al., 2022). Non-CMs, such as vascular smooth muscle cells (SMCs) and endothelial cells (ECs), play essential roles in promoting cardiovascular tissue regeneration, which is a central focus in cardiac repair and tissue engineering (Munawar and Turnbull, 2021; Tenreiro et al., 2021; Kahn-Krell et al., 2022). Advances in cell reprogramming have further enabled the generation of induced cardiovascular tissues from mouse fibroblasts, which have demonstrated reparative potential following transplantation into infarcted hearts (Cho et al., 2021). Coordinated cardiomyogenesis and neovascularization are expected to enable comprehensive heart regeneration. However, the mechanisms governing the simultaneous reprogramming of fibroblasts into multiple cardiovascular cell (CVC) lineages remain poorly understood, hindering further technical optimization. Additionally, achieving *in vivo* conversion of resident CFs into functional CVCs remains challenging due to the lack of efficient reprogramming strategies.

Our previous study demonstrated that CRISPR activation (CRISPRa) of endogenous cardiogenic genes can reprogram fibroblasts into induced cardiovascular progenitor cells possessing self-renewal capacity and differentiation potential toward CVC fates (Jiang et al., 2022). Building upon this foundation, we sought to develop a novel *in vivo* strategy for CVC regeneration by employing CRISPRa of cardiogenic genes in resident CFs. In this study, we provide the first proof of concept for *in vivo* CVC reprogramming. Specifically, we found that CRISPRa of cardiogenic genes reprogrammed a subset of CFs into CM- or SMC-like cells within the heart. Although overall cardiac function was not significantly improved, further optimization of the current approach will be necessary to enhance reprogramming efficiency and achieve effective *in vivo* CVC induction for cardiac repair.

## 2 Methods

All research protocols conformed to the Guidelines for the Care and Use of Laboratory Animals published by the National Institutes of Health (National Academies Press, eighth edition, 2011). All animal use protocols and experiments conducted in this study were approved and overseen by the University of Cincinnati Animal Care and Use Committee.

### 2.1 Mouse lines

Pdgfra^CreER^ (Stock#032770), Rosa-LSL-dCas9^ST^ (Stock#031925), Nkx2.5CE^eGFP^ (Stock#029489), and Rosa-LSL-tdTomato (Stock#007914) mice were obtained from The Jackson Laboratory. Genotyping was confirmed by standard PCR according to the manufacturer’s protocols.

### 2.2 CF isolation

Adult mouse (6–8 weeks) hearts were harvested and processed for fibroblast isolation through enzymatic digestion, as modified from our previous study (Jiang et al., 2022). The dissociated tissue suspension was further pipetted on ice, filtered through a 40 µm nylon strainer, and washed with cold DPBS to 30 mL total volume. Cells were pelleted (200×g, 15 min, 4 °C, no brake), resuspended in DMEM supplemented with 10% fetal bovine serum (FBS) and 1% penicillin-streptomycin, and plated at the desired density. To enrich for adherent fibroblasts, the culture medium was replaced after 2 h to remove non-adherent cells. Adherent cells were maintained in DMEM +10% FBS +1% penicillin-streptomycin for subsequent experiments, and the media were changed every 2–3 days. Fibroblasts were digested with 0.05% trypsin for further cell passage.

### 2.3 sgRNA vector construction

Mouse U6 promoter-driven sgRNA cassette (modified from Addgene#84832) and scFv-VP64 fragment (modified from Addgene#60904) were synthesized and constructed in an AAV backbone using the Gateway cloning system (VectorBuilder, IL). The sgRNA design, synthesis, and cloning have been previously described (Jiang et al., 2022). The detailed sequences of sgRNAs and empty backbone with scFv-VP64 are shown in the Supplementary Material.

### 2.4 AAV production

HEK293 cells (Cell Biolabs, AAV-100) were cultured in high-glucose DMEM supplemented with 10% FBS and 1% penicillin–streptomycin under standard conditions (37 °C, 5% CO_2_). Twenty-four hours before transfection, cells were plated in 10 cm dishes (Falcon) at approximately 70% confluency. For each dish, 4 µg of pAdDeltaF6 (Addgene #112867), 2 µg of pAAV2/1 (Addgene #112862), and 2 µg of the sgRNA AAV vector (scrambled target seed sequence as control) were transfected into the cells using 30 µL of Polyethyleneimine (PEI, Thermo Scientific) mixed in Opti-MEM reduced serum media (Gibco, 31985070) following the manufacturer’s protocol. After incubation overnight, the transfection medium was replaced with 10 mL of fibroblast growth medium. At 72 h post-transfection, viral supernatants were collected, filtered through 0.45 µm PVDF syringe filters (Fisher Scientific), concentrated using AAVanced™ Concentration Reagent (System Bioscience, AAV100A-1), and stored at −80 °C until further use. Quantitative PCR was used to titrate viral concentration as described (Aurnhammer et al., 2012).

### 2.5 *In vitro* cell induction

CFs were seeded onto 6-well culture dishes at an approximate density of 1 × 10^5^ cells per well in fibroblast growth medium, and each well was supplemented with 100 µL of concentrated AAV viral media for 5 days. Subsequently, CFs were cultured with reprogramming medium composed of high-glucose DMEM supplemented with 10% FBS, 1% non-essential amino acids (NEAA), 1% L-glutamine, 1% penicillin-streptomycin, 10 ng/mL bFGF, 3 µM CHIR99021 (BioTechne), 3 µM SB431542 (SelleckChem), and 10^3^ U/mL LIF (Sigma-Aldrich). The medium was refreshed every 2 days. To further enrich for reprogrammed populations and recapitulate aspects of the cardiac developmental microenvironment, a suspension culture system was employed to generate multicellular spheroids. Cells from each 6-well were dissociated and transferred into low-adhesion plates (Falcon) containing reprogramming medium to promote spheroid assembly over 2–3 days. For lineage specification, spheroids were then plated onto 0.2% gelatin-coated dishes and cultured in the appropriate differentiation media. For CM induction, attached spheroids were incubated in CM differentiation medium consisting of high-glucose DMEM supplemented with 1% NEAA, 1% L-glutamine, 1× β-mercaptoethanol, N2 supplement (Gibco), B27 supplement (Gibco), 0.1% bovine serum albumin (BSA; Sigma-Aldrich), 12.5 ng/mL bFGF, 40 ng/mL BMP4 (R&D Systems), 10 ng/mL Activin A, 15 ng/mL Wnt-3a (R&D Systems), 5 µM IWP-4 (Stemgent), 5 ng/mL VEGFA (R&D Systems), and 1% penicillin–streptomycin.

### 2.6 Quantitative PCR

Total RNA was isolated using TRIzol™ Reagent (Invitrogen) following the manufacturer’s protocol. Briefly, cells were washed twice with cold PBS, lysed directly in TRIzol reagent, and homogenized by pipetting. The lysate was incubated at room temperature for 5 min, followed by the addition of chloroform and centrifugation at 12,000 × g for 15 min at 4 °C to separate the phases. The aqueous phase containing RNA was carefully transferred to a new tube, mixed with isopropanol, and centrifuged to pellet the RNA. The RNA pellet was washed with 75% ethanol, air-dried, and dissolved in RNase-free water. RNA concentration and purity were determined using a NanoDrop™ spectrophotometer (Thermo Fisher Scientific). Complementary DNA (cDNA) was synthesized from 1 µg of total RNA using the iScript™ cDNA Synthesis Kit (Bio-Rad) following the manufacturer’s instructions. Quantitative real-time PCR (RT-qPCR) was performed using the iTaq™ Universal SYBR® Green Supermix (Bio-Rad) on a CFX96 Touch Real-Time PCR Detection System (Bio-Rad). Gene-specific primers were used to amplify target transcripts, and GAPDH served as an internal control. Relative expression levels were calculated using the 2^ΔΔCt^ method, and data were expressed as fold changes compared with control samples. All primer sequences are listed in the Supplementary Material.

### 2.7 Western blot

Cells were lysed in 1× Cell Lysis Buffer (Cell Signaling Technology, #9803) and incubated on ice for 30 min with intermittent mixing. Lysates were centrifuged at 12,000 × *g* for 15 min at 4 °C, and the clarified supernatant was collected. Protein concentrations were determined using the Bradford Protein Assay (Bio-Rad) according to the manufacturer’s protocol. Equal amounts of total protein were separated on 4%–12% NuPAGE™ Bis-Tris Midi Protein Gels (Invitrogen) and transferred onto Nitrocellulose membranes (Invitrogen). Membranes were blocked with 5% nonfat dry milk (Bio-Rad) in TBS-T (Tris-buffered saline containing 0.1% Tween-20) for 1 h at room temperature, followed by incubation with primary antibodies (diluted in TBS) overnight at 4 °C. After washing, membranes were incubated with HRP-conjugated secondary antibodies (diluted in TBS) for 1 h at room temperature. Immunoblots were visualized using SuperSignal™ West Pico PLUS Chemiluminescent Substrate (Thermo Fisher) and imaged with a ChemiDoc™ Imaging System (Bio-Rad). Protein expression levels were normalized to β-actin, and relative band intensities were quantified using ImageJ. All antibody information is provided in the Supplementary Material.

### 2.8 Immunocytochemistry

Immunofluorescence staining was performed on cells seeded onto 0.2% gelatin-coated slices in 35-mm tissue culture dish (Falcon). Cells were fixed with 4% paraformaldehyde (PFA) for 10 min and permeabilized in 0.2% Triton X-100 for 10 min. After treatment with 2% BSA blocking buffer for 30 min, samples were incubated with specific primary antibody diluted in PBS with 0.1% BSA, followed by their related secondary antibody. Following staining, samples were covered with VECTASHIELD Antifade Mounting Medium with DAPI (Vector Labs), observed under a fluorescent microscope (Olympus).

### 2.9 Mouse MI model and AAV injection

MI was induced by permanent ligation of the left anterior descending (LAD) coronary artery in 8-week-old male or female mice according to previously established procedures (Jiang et al., 2022). Briefly, mice were anesthetized via intraperitoneal injection of 0.1% ketamine and 0.02% xylazine. The heart was exposed through a left-sided minimal thoracotomy, and the LAD was ligated using a 6–0 silk suture. Ten minutes after ligation, 30 µL of the AAV-sgRNA viral suspension (approximately 1 × 10^12^ GC per heart), or control AAV was delivered into three distinct sites along the border between the infarct and peri-infarct regions. After AAV injection, the chest was sutured closed, and the mouse was allowed to recover using a ventilator and a heating pad.

### 2.10 Echocardiography

Cardiac function was assessed by transthoracic echocardiography using a VisualSonics Vevo 2100 Imaging System equipped with a 15 MHz transducer, as described previously. Mice were positioned supine on a temperature-controlled heating pad (37 °C) and maintained under light isoflurane anesthesia (1.5% isoflurane in 98.5% oxygen) during imaging. Two-dimensional guided M-mode echocardiographic recordings were obtained from the parasternal short-axis view at the level perpendicular to the LV anterior and posterior walls. Left ventricular end-diastolic diameter (LVDd) and end-systolic diameter (LVDs) were measured from M-mode tracings. Ejection fraction (EF) and fractional shortening (FS) were then calculated according to the following formulas: EF = [(LVDd^3^ - LVDs^3^)/LVDd^3^] × 100% and FS = [(LVDd - LVDs)/LVDd] × 100%. Hemodynamic parameters, including stroke volume (SV), heart rate (HR), and cardiac output (CO), were quantified via transthoracic echocardiography using the integrated analysis software of the VisualSonics Vevo 2100 system. All echocardiographic measurements followed the American Society for Echocardiography leading-edge methodology and were averaged over at least three consecutive cardiac cycles.

### 2.11 Immunohistochemistry

Heart tissues were collected at 8 weeks, fixed in 4% PFA overnight at 4 °C, and then incubated in 30% sucrose for 24 h at 4 °C. The fixed samples were embedded in OCT compound and rapidly frozen in liquid nitrogen. Frozen tissues were sectioned at 8 µm thickness using a cryostat maintained at −20 °C and mounted onto Polysine slides. Sections were treated with 0.1% protein kinase A (PKA) solution for 5 min, followed by incubation in 0.1% Triton X-100 with serum blocking buffer for 10 min. After washing with PBS, the slides were incubated with primary antibodies (diluted in PBS with 0.1% BSA) overnight at 4 °C. Following removal of the primary antibodies, fluorescein-conjugated secondary antibodies were applied for 1 h at room temperature. Fluorescence was checked under the fluorescence microscope (Olympus), and then nuclei were stained with DAPI using the VECTASHIELD Antifade Mounting Medium. All staining was manually counted under the microscope and quantified using ImageJ. For each mouse, four to six fields from the infarct and border zones per section were analyzed to quantify CMs and SMCs. Reprogramming efficiency was expressed as the percentage of tdTomato^+^ cells among the CMs or SMCs in these fields.

### 2.12 Statistical analysis

All statistical analyses were performed using GraphPad Prism version 10 (GraphPad Software, Inc.). Comparisons between two groups were conducted using an unpaired Student’s t-test, while multiple group comparisons were analyzed by one or two-way ANOVA followed by Bonferroni *post hoc* testing. Data are expressed as mean ± standard deviation (SD), and a *p* value <0.05 was considered statistically significant.

## 3 Results

### 3.1 CRISPR-mediated overexpression of endogenous cardiogenic genes in CFs

**FIGURE 1:**
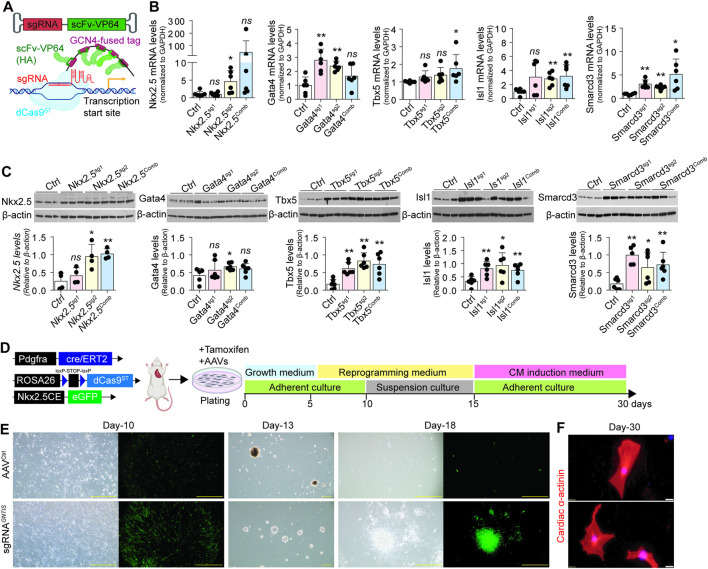
Activation of endogenous cardiogenic genes for fibroblast reprogramming. **(A)** Schematic of the CRISPRa system targeting the transcription start site of cardiogenic genes. The scFv (single-chain variable fragment) was engineered to recognize and bind the GCN4 peptide for transcriptional activation. **(B,C)** Quantitative PCR **(B)** and Western blot **(C)** analyses of cardiogenic gene expression in CFs isolated from tamoxifen-treated Pdgfra^creERT2^;Rosa-LSL-dCas9^ST^ transgenic mice after 7 days of transfection with AAVs carrying individual sgRNAs. Data are presented as mean ± standard error (n = 6 per group); Versus scramble sgRNA control: ^ns^, not significant; *p < 0.05; **p < 0.01. **(D)** Schematic illustration of the experimental design using CFs from Pdgfra^creERT2^;Rosa-LSL-dCas9^ST^;Nkx2.5CE^eGFP^ mice, showing the induction procedure following tamoxifen treatment and infection with the AAV cocktail expressing sgRNAs. **(E)** Morphological changes in CRISPRa-induced and control CFs before and after suspension culture. Green fluorescence indicates activation of the Nkx2.5^CEeGFP^ reporter. Scale bars, 200 µm. **(F)** Representative immunostaining images for cardiac markers in CRISPRa^*GNTIS*^-induced cells after suspension culture and reattachment. Scale bars, 20 µm.

The dCas9 modified with GCN4 peptides (SunTag, dCas9^ST^) can specifically activate single-guide RNA (sgRNA)-targeted genes by recruiting the fused scFv-VP64 transcriptional activator (Tanenbaum et al., 2014). To streamline gene delivery, we utilized the Rosa-LSL-dCas9^ST^ transgene model (Wangensteen et al., 2018) and constructed adeno-associated viral vectors (AAVs) to co-express sgRNAs and scFv-VP64 (Figure 1A). Subsequently, we re-screened sgRNAs targeting critical cardiogenic genes (*Gata4, Nkx2.5, Tbx5, Isl1*, and *Smarcd3* (collectively abbreviated as *GNTIS*)) using the AAV1 system, following our previous optimization with lentiviral vectors (Jiang et al., 2022). Two sgRNAs were designed per gene and transfected into CFs isolated from tamoxifen-treated Pdgfra^creERT2^;Rosa-LSL-dCas9^ST^ mice. Gene activation was assessed by qPCR and Western blotting after 7 days of AAV transfection (Figures 1B,C). Among the tested candidates, *Nkx2.5*_sgRNA2, *Gata4*_sgRNA2, *Isl1*_sgRNA2, *Smarcd3*_sgRNA1, and *Tbx5*_sgRNA1/2 consistently and robustly upregulated their respective target genes at both the mRNA and protein levels, exhibiting minimal variability. These were pooled to generate the sgRNA^*GNTIS*^ cocktail for downstream studies.

To evaluate the reprogramming potential of *GNTIS* activation, CFs were isolated from tamoxifen-treated Pdgfra^creERT2^;Rosa-LSL-dCas9^ST^;Nkx2.5CE (cardiac enhancer)^eGFP^ mice (Figure 1D). Transfection with AAVs carrying the pooled sgRNA^*GNTIS*^ cocktail robustly activated Nkx2.5CE^eGFP^ expression after 10 days (Figure 1E). During suspension culture, CFs receiving the sgRNA^*GNTIS*^ cocktail progressively aggregated and formed compact spheroid structures by day 13. In contrast, CFs transfected with control AAVs aggregated into non-proliferative cell clusters lacking the ability to replate. Upon replating, the sgRNA^*GNTIS*^-induced spheroids retained Nkx2.5CE^eGFP^ expression on day 18. When cultured in CM differentiation medium, a subset of cells exhibited expression of the cardiac structural protein α-actinin (Figure 1F). Collectively, these findings indicate that CRISPRa-mediated activation of *GNTIS* genes reprograms CFs toward the cardiac-like lineage via an intermediate stage, consistent with our previous observations (Jiang et al., 2022).

### 3.2 Targeted CRISPRa in CF bolsters hemodynamic output without improving global systolic function

**FIGURE 2:**
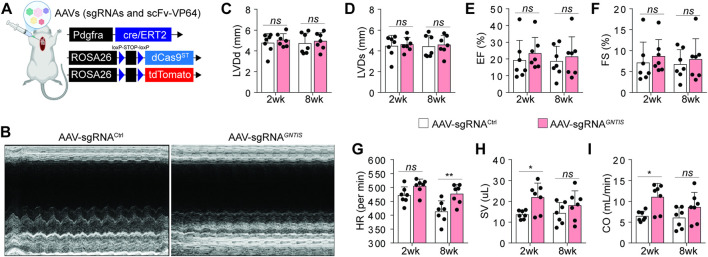
Assessment of cardiac function in MI mice. **(A)** Schematic of Pdgfra^CreERT2^;Rosa-LSL-dCas9^ST^;Rosa-LSL-tdTomato mice used to generate the MI model and for intramyocardial injection of AAVs expressing sgRNAs and scFv-VP64 during surgery. **(B)** Representative imaging of M-mode echocardiography at 8 weeks after surgery. **(C–I)** Quantification of cardiac function, including **(C)** left ventricular end-diastolic dimension (LVDd), **(D)** left ventricular end-systolic dimension (LVDs), **(E)** ejection fraction (EF), **(F)** fractional shortening (FS), **(G)** heart rate (HR), **(H)** stroke volume (SV), and **(I)** cardiac output (CO) measured at 2 or 8 weeks post-MI. Data are presented as mean ± standard error (n = 7 per group); ^ns^, not significant; *p < 0.05.

To investigate CF fate after MI, we generated Pdgfra^CreERT2^;Rosa-LSL-dCas9^ST^;Rosa-LSL-tdTomato mice and administered tamoxifen before MI induction. The mice were subsequently injected with the AAV cocktail carrying either sgRNA^*GNTIS*^ or sgRNA^Ctrl^ together with scFv-VP64 (Figure 2A). Cardiac function was evaluated by echocardiography. Unexpectedly, MI mice injected with either sgRNA^*GNTIS*^ or sgRNA^Ctrl^ exhibited comparable cardiac function, with no significant differences in LVDd, LVDs, EF, or FS during 2–8 weeks post-MI (Figures 2B–F). On the other hand, we examined additional hemodynamic parameters. Heart rate, stroke volume, and cardiac output were significantly higher in the sgRNA^*GNTIS*^ group compared to the sgRNA^Ctrl^ at the 2 or 8-week post-MI (Figures 2G–I). Thus, CRISPRa of *GNTIS* (CRISPRa^*GNTIS*^) in CFs modulated cardiac output dynamics without evidence of enhanced intrinsic systolic performance in MI mice.

### 3.3 *In vivo* CRISPRa induces CF transdifferentiation toward cardiovascular lineages

**FIGURE 3:**
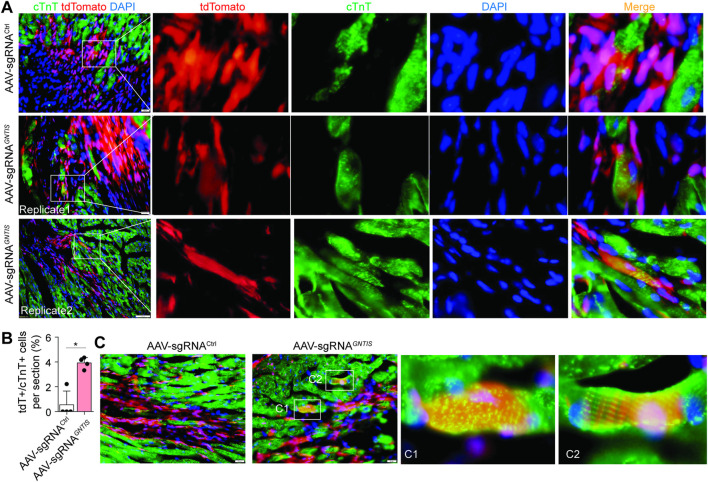
Reprogramming potential of CFs into CM-like cells. **(A)** Representative images of cTnT^+^ CM-like cells derived from tdTomato^+^ CFs at 8 weeks post-MI. The infarct areas are highlighted. Scale bars, 20 µm. **(B)** Quantification of tdTomato^+^ percentage in the total cTnT^+^ CMs. Data are presented as mean ± standard error (n = 4 per group). See also Supplementary Figure S1. **(C)** Representative images of α-actinin^+^ CM-like cells derived from tdTomato^+^ CFs at 8 weeks post-MI. Magnified regions are presented in panels C1 and C2. Scale bars, 20 µm.

Mouse hearts were harvested 8 weeks after MI. No significant difference in infarct size was observed between the sgRNA^Ctrl^ and sgRNA^*GNTIS*^ groups (data not shown), consistent with their comparable cardiac function (Figures 2B–F). Immunostaining analysis revealed that approximately 4% of cTnT^+^ CMs in the infarct region originated from tdTomato^+^ CFs in the sgRNA^*GNTIS*^ group, whereas only a rare number of tdTomato^+^/cTnT^+^ double-positive cells were detected in sgRNA^Ctrl^ hearts (Figures 3A,B; Supplementary Figure S1). Notably, most of these reprogrammed cells were located adjacent to, or interspersed among, existing CMs in the border zone, suggesting a potential contribution to cell-cell coupling. A subset of cells reprogrammed from tdTomato^+^ CFs expressed sarcomeric α-actinin (Figure 3C) and exhibited either less organized (C1 panel) or well-organized (C2 panel) striated structures, suggesting different maturation stages, including intermediate states.

**FIGURE 4:**
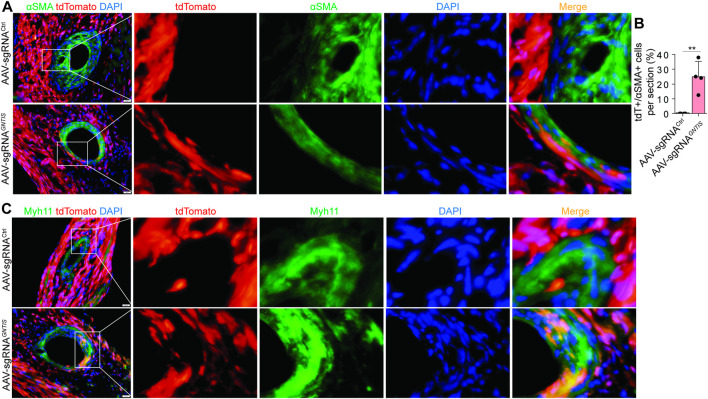
Reprogramming potential of CFs into SMC-like cells. **(A)** Representative images of αSMA^+^ SMC-like cells derived from tdTomato^+^ CFs at 8 weeks post-MI. The vessel wall regions are highlighted. Scale bars, 20 µm. **(B)** Quantification of tdTomato^+^ percentage in the total αSMA^+^ SMCs. Data are presented as mean ± standard error (n = 4 per group). **(C)** Representative images of SM-MHC (or Myh11)^+^ cells derived from tdTomato^+^ CFs at 8 weeks post-MI. The vessel wall regions are highlighted. Scale bars, 20 µm. See also Supplementary Figure S2.

The potential for reprogramming into vascular cells was also evaluated. Approximately 20% of αSMA^+^ SMCs in vessel walls were derived from tdTomato^+^ CFs in the sgRNA^*GNTIS*^ group, while no tdTomato^+^/αSMA^+^ cells were identified in the sgRNA^Ctrl^ group (Figures 4A,B; Supplementary Figure S2). The CF to SMC transdifferentiation was also verified by the expression of smooth-muscle myosin heavy chain (SM-MHC or Myh11) in tdTomato^+^ CFs (Figure 4C). Importantly, these reprogrammed cells were predominantly integrated into the vessel wall within the infarct zone, which was enriched in highly activated tdTomato^+^ CFs. Under the current conditions, very few tdTomato^+^ CFs were reprogrammed into endothelial cells lining the vessel lumen (data not shown).

## 4 Discussion

CFs, which dominate the post-MI microenvironment, represent an accessible target for regenerative reprogramming. Converting these resident CFs into CVCs, comprising CMs and vascular cells, could establish a new framework for heart regeneration. In this study, we combined a CRISPRa transgenic model with an AAV delivery system to activate the endogenous cardiogenic gene network *GNTIS* within CFs. Activation of *GNTIS* drove CFs into an intermediate state with an active *Nkx2.5* cardiac enhancer, indicative of cardiogenic potential. In the MI context, this CRISPRa-mediated *GNTIS* activation induced partial transdifferentiation of CFs toward CM and SMC fates. Although the efficiency was insufficient to restore cardiac function, our results provide the first proof of concept for direct reprogramming of CFs into CVCs *in vivo*. Continued refinement of this approach and deeper mechanistic insight will be critical to developing effective strategies for heart repair involving multiple regenerative lineages.

We selected CRISPRa targeting of *GNTIS* based on our previous studies using tail tip fibroblasts (TTFs) (Jiang et al., 2022). We further hypothesized that activation of these targets could reconstruct the cardiogenic gene network, thereby recapitulating heart developmental pathways and initiating lineage reprogramming (Jiang et al., 2022). Upon transitioning to the non-integrative AAV system, *GNTIS* activation reached an acceptable level at both mRNA and protein scales, promoting CFs toward a precursor-like state characterized by *Nkx2.5* cardiac enhancer activity and CM transdifferentiation potential. Interestingly, Gata4 and Nkx2.5 were detected in a subset of normal CFs, showing basal protein expression even in negative controls. Several cardiogenic genes such as *Gata4, Tbx20*, and *Hand2* are specifically expressed in CFs, potentially contributing to cardiac homeostasis and pathology, while absent in TTFs (Furtado et al., 2014). It is noteworthy that dCas9 activators preferentially induce lowly expressed genes with higher fold changes compared to highly expressed ones, due to their reliance on endogenous transcriptional machinery (Chavez et al., 2016). Therefore, unlike in TTFs, cardiogenic genes already active in CFs may reach transcriptional saturation upon dCas9-mediated activation, which can be verified by comparing their transcriptional levels to those in cell counterparts. These observations may explain why CRISPRa targeting of *GNTIS* genes yielded suboptimal reprogramming efficiency in CFs, suggesting further optimization is needed.

Despite the absence of overt structural repair or full functional recovery, *in vivo* CRISPRa-mediated activation of *GNTIS* genes in CFs influenced global hemodynamic performance, as evidenced by increased heart rate, stroke volume, and cardiac output. Notably, an elevation in either heart rate or stroke volume alone is sufficient to augment cardiac output, even in the absence of changes in systolic indices such as EF or FS (Patel et al., 2021). Moreover, since EF can be defined as the ratio of stroke volume to end-diastolic volume, proportional increases in both parameters may preserve EF despite augmented forward flow (Marwick, 2018). In this context, the observed increase in SV may reflect preload-related or volume-mediated adaptations rather than enhanced intrinsic contractility. It is conceivable that CRISPRa^*GNTIS*^ partially modulates the fibrotic program of CFs, potentially reducing myocardial stiffness and improving ventricular compliance, while this mechanistic link requires further experimental validation.

Our study further demonstrated the plasticity of CFs undergoing reprogramming into CVC-like types *via* CRISPRa^*GNTIS*^, although the reprogramming efficiency remained relatively low. After excluding potential cell fusion events, we observed that only a small fraction of newly formed CMs were derived from CF reprogramming, suggesting a limited contribution to cardiomyogenesis. In contrast, a more substantial CF-to-SMC conversion was detected. This preferential transdifferentiation toward the SMC lineage may provide partial functional compensation, potentially through participation in or facilitation of post-MI neovascular remodeling. These αSMA or SM-MHC-positive reprogrammed cells predominantly localized to the vessel wall rather than the luminal surface, indicating SMC characteristics with minimal endothelial contribution. Notably, αSMA, a shared marker of myofibroblasts and SMCs, was markedly reduced in the myocardial interstitium but retained in vascular cells several weeks post-MI, consistent with prior fibroblast lineage-tracing findings (Fu et al., 2018). Furthermore, spontaneous CF-to-SMC transdifferentiation was not observed under pathological stress in the control group, reinforcing the concept of induced SMC reprogramming from fibroblasts (Hirai et al., 2018). Thus, robust *in vivo* SMC reprogramming may serve as a priming strategy for cardiac regeneration through enhanced neovascularization and arteriogenesis (Jung et al., 2025).

The precise mechanisms governing CF lineage conversion following CRISPRa^*GNTIS*^ remain incompletely understood. Based on our previous *in vitro* CRISPR-induced reprogramming model (Jiang et al., 2022), fibroblasts first transitioned into precursor-like intermediate states before committing toward either CM or SMC lineages. Such bifurcation appeared to be influenced by local environmental cues, reminiscent of signaling pathways employed during directed differentiation of pluripotent stem cells into CMs or SMCs (Shen et al., 2021; He et al., 2022). Developmental studies further support this possibility. The initiation of Nkx2.5 expression marks commitment to cardiovascular precursor populations capable of generating both myocardial and smooth muscle lineages (Wu et al., 2006). Moreover, co-expression of Flk1 within Isl1^+^ progenitors has been shown to bias differentiation toward SMC precursors (Moretti et al., 2006). These regulatory interactions may similarly influence lineage specification following *GNTIS* gene activation. Future studies employing single-cell transcriptomic profiling coupled with sgRNA barcoding (Chardon et al., 2024) could systematically resolve the gene regulatory networks underlying CM versus SMC commitment. Integration with spatial transcriptomics would further enable dissection of the *in vivo* microenvironmental signals shaping fate decisions within the infarct niche.

Additionally, this study presents several technical limitations. In the present study, it was not feasible to employ an all-in-one AAV construct co-expressing all sgRNA^*GNTIS*^ components and the scFv-VP64 activator due to packaging constraints. As a result, individual sgRNAs within the AAV cocktail may have been delivered or expressed unevenly across target cells, potentially generating heterogeneous levels of transcriptional activation. It is therefore plausible that differences in gene dosage or activation stoichiometry contributed to divergent lineage outcomes, consistent with prior reports demonstrating dosage-dependent effects during induced CM reprogramming (Wang et al., 2015). Such heterogeneity may result in subthreshold or incomplete transcriptional activation in a substantial fraction of cells, thereby limiting measurable effects on global ventricular remodeling parameters. While the AAV1 serotype used in this study performed efficiently *in vitro*, its capsid tropism may be suboptimal for targeting CFs *in vivo*, given the cellular heterogeneity of the heart. Because CFs are activated and proliferate rapidly in the infarct zone, non-integrative AAVs can only transfect a subset of cells at the time of injection. As a result, the majority of fibrotic descendants, myofibroblasts, and subsequently activated CFs would not carry the reprogramming components, thereby reducing the initial pool of reprogrammable cells and complicating quantification of transduction or reprogramming efficiency. Future improvements in vector design, dCas9 activator engineering, and the identification of more potent sgRNAs will be essential to advance the *in vivo* CRISPRa strategy for targeted reprogramming of CFs into specific cardiovascular lineages.

In summary, we utilized CRISPRa-mediated activation of cardiogenic genes to achieve the first *in vivo* proof of concept for reprogramming CF plasticity into CVC-like lineages, including CMs and SMCs. This study demonstrates the feasibility of directly inducing CVC lineages within the injured heart through transcriptional reprogramming, offering an innovative route for regenerative therapy.

## Data Availability

The original contributions presented in the study are included in the article/Supplementary Material, further inquiries can be directed to the corresponding authors.
