# Supplementary material for: In vivo CRISPR-mediated activation of cardiogenic genes to reprogram cardiac fibroblasts
Source: Front Cell Dev Biol. 2026 Apr 22;14:1812941. doi: 10.3389/fcell.2026.1812941 (PMC13143979; doi:10.3389/fcell.2026.1812941)
Supplement: Supplementary file 1 [file Table1.docx]

**Table S1. Vector construction**

| Oligonucleotides for the sgRNA construct | |
| --- | --- |
| Target | Sequences (5’-3’) |
| *Nkx2.5* | sgRNA_1: CACCGGAGTCTAAACGCAGGGTTGG  Reverse complement: AAACCCAACCCTGCGTTTAGACTCC |
|  | sgRNA_2: CACCGAGCAATCTCCTTGCAGGAGT  Reverse complement: AAACACTCCTGCAAGGAGATTGCTC |
| *Gata4* | sgRNA_1: CACCGGCAGCAAACCGCAAGGACGT  Reverse complement: AAACACGTCCTTGCGGTTTGCTGCC |
|  | sgRNA_2: CACCGGGGGCGGGGGAGCCCGGACC  Reverse complement: AAACGGTCCGGGCTCCCCCGCCCCC |
| *Tbx5* | sgRNA_1: CACCGGGGTATATCTAGTGAGAAAG  Reverse complement: AAACCTTTCTCACTAGATATACCCC |
|  | sgRNA_2: CACCGAGTCAACAGAGATGGAAGGA  Reverse complement: AAACTCCTTCCATCTCTGTTGACTC |
| *Smarcd3* | sgRNA_1: CACCGGACCACGCGGAGTGCACAGG  Reverse complement: AAACCCTGTGCACTCCGCGTGGTCC |
|  | sgRNA_2: CACCGGCAGGCAGACCCCGGGCCGC  Reverse complement: AAACGCGGCCCGGGGTCTGCCTGCC |
| *Isl1* | sgRNA_1: CACCGGGAGGCTTAATCCTCTCCTG  Reverse complement: AAACCAGGAGAGGATTAAGCCTCCC |
|  | sgRNA_2: CACCGCTCCTGCGGGCTCGCCGAGC  Reverse complement: AAACGCTCGGCGAGCCCGCAGGAGC |
| Sequence (5’-3’) of sgRNA-expressing AAV backbone (empty) | |
| U6-sgRNA scaffold-EF1A-scFV-VP64:  CCTGCAGGCAGCTGCGCGCTCGCTCGCTCACTGAGGCCGCCCGGGCAAAGCCCGGGCGTCGGGCGACCTTTGGTCGCCCGGCCTCAGTGAGCGAGCGAGCGCGCAGAGAGGGAGTGGCCAACTCCATCACTAGGGGTTCCTTCTAGACAACTTTGTATAGAAAAGTTGGAGGGCCTATTTCCCATGATTCCTTCATATTTGCATATACGATACAAGGCTGTTAGAGAGATAATTGGAATTAATTTGACTGTAAACACAAAGATATTAGTACAAAATACGTGACGTAGAAAGTAATAATTTCTTGGGTAGTTTGCAGTTTTAAAATTATGTTTTAAAATGGACTATCATATGCTTACCGTAACTTGAAAGTATTTCGATTTCTTGGCTTTATATATCTTGTGGAAAGGACGAAACACCTTCGGCAGGTGGTCGTTTTACAACGTCGTGACTGGGAAAACCCTGGCGTTACCCAACTTAATCGCCTTGCAGCACATCCCCCTTTCGCCAGCTGGCGTAATAGCGAAGAGGCCCGCACCGATCGCCCTTCCCAACAGTTGCGCAGCCTGAACGGCGAGTGGCGCTTTGCCTGGTTTCCGGCACCAGAAGCGGTGCCGGAAAGCTGGCTGGAGTGCGATCTTCCTGAGGCCGATACTGTCGTCGTCCCCTCAAACTGGCAGCACCTGCACCGGTTTTAGAGCTAGAAATAGCAAGTTAAAATAAGGCTAGTCCGTTATCAACTTGAAAAAGTGGCACCGAGTCGGTGCTTTTTTCAAGTTTGTACAAAAAAGCAGGCTGGCTCCGGTGCCCGTCAGTGGGCAGAGCGCACATCGCCCACAGTCCCCGAGAAGTTGGGGGGAGGGGTCGGCAATTGAACCGGTGCCTAGAGAAGGTGGCGCGGGGTAAACTGGGAAAGTGATGTCGTGTACTGGCTCCGCCTTTTTCCCGAGGGTGGGGGAGAACCGTATATAAGTGCAGTAGTCGCCGTGAACGTTCTTTTTCGCAACGGGTTTGCCGCCAGAACACAGGTAAGTGCCGTGTGTGGTTCCCGCGGGCCTGGCCTCTTTACGGGTTATGGCCCTTGCGTGCCTTGAATTACTTCCACCTGGCTGCAGTACGTGATTCTTGATCCCGAGCTTCGGGTTGGAAGTGGGTGGGAGAGTTCGAGGCCTTGCGCTTAAGGAGCCCCTTCGCCTCGTGCTTGAGTTGAGGCCTGGCCTGGGCGCTGGGGCCGCCGCGTGCGAATCTGGTGGCACCTTCGCGCCTGTCTCGCTGCTTTCGATAAGTCTCTAGCCATTTAAAATTTTTGATGACCTGCTGCGACGCTTTTTTTCTGGCAAGATAGTCTTGTAAATGCGGGCCAAGATCTGCACACTGGTATTTCGGTTTTTGGGGCCGCGGGCGGCGACGGGGCCCGTGCGTCCCAGCGCACATGTTCGGCGAGGCGGGGCCTGCGAGCGCGGCCACCGAGAATCGGACGGGGGTAGTCTCAAGCTGGCCGGCCTGCTCTGGTGCCTGGTCTCGCGCCGCCGTGTATCGCCCCGCCCTGGGCGGCAAGGCTGGCCCGGTCGGCACCAGTTGCGTGAGCGGAAAGATGGCCGCTTCCCGGCCCTGCTGCAGGGAGCTCAAAATGGAGGACGCGGCGCTCGGGAGAGCGGGCGGGTGAGTCACCCACACAAAGGAAAAGGGCCTTTCCGTCCTCAGCCGTCGCTTCATGTGACTCCACGGAGTACCGGGCGCCGTCCAGGCACCTCGATTAGTTCTCGAGCTTTTGGAGTACGTCGTCTTTAGGTTGGGGGGAGGGGTTTTATGCGATGGAGTTTCCCCACACTGAGTGGGTGGAGACTGAAGTTAGGCCAGCTTGGCACTTGATGTAATTCTCCTTGGAATTTGCCCTTTTTGAGTTTGGATCTTGGTTCATTCTCAAGCCTCAGACAGTGGTTCAAAGTTTTTTTCTTCCATTTCAGGTGTCGTGAACCCAGCTTTCTTGTACAAAGTGGGCCACCATGGGCCCCGACATCGTGATGACCCAGAGCCCCAGCAGCCTGAGCGCCAGCGTGGGCGACCGCGTGACCATCACATGCCGCAGCAGCACCGGCGCCGTGACCACCAGCAACTACGCCAGCTGGGTGCAGGAGAAGCCCGGCAAGCTGTTCAAGGGCCTGATCGGCGGCACCAACAACCGCGCCCCCGGCGTGCCCAGCCGCTTCAGCGGCAGCCTGATCGGCGACAAGGCCACCCTGACCATCAGCAGCCTGCAGCCCGAGGACTTCGCCACCTACTTCTGCGCCCTGTGGTACAGCAACCACTGGGTGTTCGGCCAGGGCACCAAGGTGGAGCTGAAGCGCGGCGGCGGCGGCAGCGGCGGCGGCGGCAGCGGCGGCGGCGGCAGCAGCGGCGGCGGCAGCGAGGTGAAGCTGCTGGAGAGCGGCGGCGGCCTGGTGCAGCCCGGCGGCAGCCTGAAGCTGAGCTGCGCCGTGAGCGGCTTCAGCCTGACCGACTACGGCGTGAACTGGGTGCGCCAGGCCCCCGGCCGCGGCCTGGAGTGGATCGGCGTGATCTGGGGCGACGGCATCACCGACTACAACAGCGCCCTGAAGGACCGCTTCATCATCAGCAAGGACAACGGCAAGAACACCGTGTACCTGCAGATGAGCAAGGTGCGCAGCGACGACACCGCCCTGTACTACTGCGTGACCGGCCTGTTCGACTACTGGGGCCAGGGCACCCTGGTGACCGTGAGCAGCTACCCATACGATGTTCCAGATTACGCTGGTGGAGGCGGAGGTTCTGGGGGAGGAGGTAGTGGCGGTGGTGGTTCAGGAGGCGGCGGAAGCTTGGATCCAGGTGGAGGTGGAAGCGGTGATGCTTTAGACGATTTTGACTTAGATATGCTTGGTTCAGACGCGTTAGACGACTTCGACCTAGACATGTTAGGCTCAGATGCATTGGACGACTTCGATTTAGATATGTTGGGCTCCGATGCCCTAGATGACTTTGATCTAGATATGCTAGGTAGTGGAGGAGGATCTCGGACCGAAGAGTACAAGCTTATCCTGAACGGTAAAACCCTGAAAGGTGAAACCACCACCGAAGCTGTTGACGCTGCTACCGCGGAAAAAGTTTTCAAACAGTACGCTAACGACAACGGTGTTGACGGTGAATGGACCTACGACGACGCTACCAAAACCTTCACGGTAACCGAAGGTGGTGGTAGCGGTGGTGGTACTAGTCCAAAAACAAGGAGGAGACCGCGAAGATCACAACGGAAAAGGCCGCCTACGCCATGGCCGTAACAACTTTATTATACATAGTTGGAATTCCGATAATCAACCTCTGGATTACAAAATTTGTGAAAGATTGACTGGTATTCTTAACTATGTTGCTCCTTTTACGCTATGTGGATACGCTGCTTTAATGCCTTTGTATCATGCTATTGCTTCCCGTATGGCTTTCATTTTCTCCTCCTTGTATAAATCCTGGTTGCTGTCTCTTTATGAGGAGTTGTGGCCCGTTGTCAGGCAACGTGGCGTGGTGTGCACTGTGTTTGCTGACGCAACCCCCACTGGTTGGGGCATTGCCACCACCTGTCAGCTCCTTTCCGGGACTTTCGCTTTCCCCCTCCCTATTGCCACGGCGGAACTCATCGCCGCCTGCCTTGCCCGCTGCTGGACAGGGGCTCGGCTGTTGGGCACTGACAATTCCGTGGTGTTGTCGGGGAAGCTGACGTCCTTTCCATGGCTGCTCGCCTGTGTTGCCACCTGGATTCTGCGCGGGACGTCCTTCTGCTACGTCCCTTCGGCCCTCAATCCAGCGGACCTTCCTTCCCGCGGCCTGCTGCCGGCTCTGCGGCCTCTTCCGCGTCTTCGCCTTCGCCCTCAGACGAGTCGGATCTCCCTTTGGGCCGCCTCCCCGCATCGGGAATTCCTAGAGCTCGCTGATCAGCCTCGACTGTGCCTTCTAGTTGCCAGCCATCTGTTGTTTGCCCCTCCCCCGTGCCTTCCTTGACCCTGGAAGGTGCCACTCCCACTGTCCTTTCCTAATAAAATGAGGAAATTGCATCGCATTGTCTGAGTAGGTGTCATTCTATTCTGGGGGGTGGGGTGGGGCAGGACAGCAAGGGGGAGGATTGGGAAGAGAATAGCAGGCATGCTGGGGAGGGCCGCAGGAACCCCTAGTGATGGAGTTGGCCACTCCCTCTCTGCGCGCTCGCTCGCTCACTGAGGCCGGGCGACCAAAGGTCGCCCGACGCCCGGGCTTTGCCCGGGCGGCCTCAGTGAGCGAGCGAGCGCGCAGCTGCCTGCAGGGGCGCCTGATGCGGTATTTTCTCCTTACGCATCTGTGCGGTATTTCACACCGCATACGTCAAAGCAACCATAGTACGCGCCCTGTAGCGGCGCATTAAGCGCGGCGGGGGTGGTGGTTACGCGCAGCGTGACCGCTACACTTGCCAGCGCCTTAGCGCCCGCTCCTTTCGCTTTCTTCCCTTCCTTTCTCGCCACGTTCGCCGGCTTTCCCCGTCAAGCTCTAAATCGGGGGCTCCCTTTAGGGTTCCGATTTAGTGCTTTACGGCACCTCGACCCCAAAAAACTTGATTTGGGTGATGGTTCACGTAGTGGGCCATCGCCCTGATAGACGGTTTTTCGCCCTTTGACGTTGGAGTCCACGTTCTTTAATAGTGGACTCTTGTTCCAAACTGGAACAACACTCAACTCTATCTCGGGCTATTCTTTTGATTTATAAGGGATTTTGCCGATTTCGGTCTATTGGTTAAAAAATGAGCTGATTTAACAAAAATTTAACGCGAATTTTAACAAAATATTAACGTTTACAATTTTATGGTGCACTCTCAGTACAATCTGCTCTGATGCCGCATAGTTAAGCCAGCCCCGACACCCGCCAACACCCGCTGACGCGCCCTGACGGGCTTGTCTGCTCCCGGCATCCGCTTACAGACAAGCTGTGACCGTCTCCGGGAGCTGCATGTGTCAGAGGTTTTCACCGTCATCACCGAAACGCGCGAGACGAAAGGGCCTCGTGATACGCCTATTTTTATAGGTTAATGTCATGATAATAATGGTTTCTTAGACGTCAGGTGGCACTTTTCGGGGAAATGTGCGCGGAACCCCTATTTGTTTATTTTTCTAAATACATTCAAATATGTATCCGCTCATGAGACAATAACCCTGATAAATGCTTCAATAATATTGAAAAAGGAAGAGTATGAGTATTCAACATTTCCGTGTCGCCCTTATTCCCTTTTTTGCGGCATTTTGCCTTCCTGTTTTTGCTCACCCAGAAACGCTGGTGAAAGTAAAAGATGCTGAAGATCAGTTGGGTGCACGAGTGGGTTACATCGAACTGGATCTCAACAGCGGTAAGATCCTTGAGAGTTTTCGCCCCGAAGAACGTTTTCCAATGATGAGCACTTTTAAAGTTCTGCTATGTGGCGCGGTATTATCCCGTATTGACGCCGGGCAAGAGCAACTCGGTCGCCGCATACACTATTCTCAGAATGACTTGGTTGAGTACTCACCAGTCACAGAAAAGCATCTTACGGATGGCATGACAGTAAGAGAATTATGCAGTGCTGCCATAACCATGAGTGATAACACTGCGGCCAACTTACTTCTGACAACGATCGGAGGACCGAAGGAGCTAACCGCTTTTTTGCACAACATGGGGGATCATGTAACTCGCCTTGATCGTTGGGAACCGGAGCTGAATGAAGCCATACCAAACGACGAGCGTGACACCACGATGCCTGTAGCAATGGCAACAACGTTGCGCAAACTATTAACTGGCGAACTACTTACTCTAGCTTCCCGGCAACAATTAATAGACTGGATGGAGGCGGATAAAGTTGCAGGACCACTTCTGCGCTCGGCCCTTCCGGCTGGCTGGTTTATTGCTGATAAATCTGGAGCCGGTGAGCGTGGAAGCCGCGGTATCATTGCAGCACTGGGGCCAGATGGTAAGCCCTCCCGTATCGTAGTTATCTACACGACGGGGAGTCAGGCAACTATGGATGAACGAAATAGACAGATCGCTGAGATAGGTGCCTCACTGATTAAGCATTGGTAACTGTCAGACCAAGTTTACTCATATATACTTTAGATTGATTTAAAACTTCATTTTTAATTTAAAAGGATCTAGGTGAAGATCCTTTTTGATAATCTCATGACCAAAATCCCTTAACGTGAGTTTTCGTTCCACTGAGCGTCAGACCCCGTAGAAAAGATCAAAGGATCTTCTTGAGATCCTTTTTTTCTGCGCGTAATCTGCTGCTTGCAAACAAAAAAACCACCGCTACCAGCGGTGGTTTGTTTGCCGGATCAAGAGCTACCAACTCTTTTTCCGAAGGTAACTGGCTTCAGCAGAGCGCAGATACCAAATACTGTTCTTCTAGTGTAGCCGTAGTTAGGCCACCACTTCAAGAACTCTGTAGCACCGCCTACATACCTCGCTCTGCTAATCCTGTTACCAGTGGCTGCTGCCAGTGGCGATAAGTCGTGTCTTACCGGGTTGGACTCAAGACGATAGTTACCGGATAAGGCGCAGCGGTCGGGCTGAACGGGGGGTTCGTGCACACAGCCCAGCTTGGAGCGAACGACCTACACCGAACTGAGATACCTACAGCGTGAGCTATGAGAAAGCGCCACGCTTCCCGAAGGGAGAAAGGCGGACAGGTATCCGGTAAGCGGCAGGGTCGGAACAGGAGAGCGCACGAGGGAGCTTCCAGGGGGAAACGCCTGGTATCTTTATAGTCCTGTCGGGTTTCGCCACCTCTGACTTGAGCGTCGATTTTTGTGATGCTCGTCAGGGGGGCGGAGCCTATGGAAAAACGCCAGCAACGCGGCCTTTTTACGGTTCCTGGCCTTTTGCTGGCCTTTTGCTCACATGT | |

**Table S2. qPCR primers**

| Target | Sequences (5’-3’) |
| --- | --- |
| *Gapdh* | F: TGCACCACCAACTGCTTAG  R: GGATGCAGGGATGATGTTC |
| *Gata4* | F: TTCTGGGAAACTGGAGCTGG  R: TGCTTTCTGCCTGCTACACA |
| *Nkx2.5* | F: TGGGACCTTTCTCCGATCCA |
|  | R: TCCCGGTCCTAGTGTGGAAT |
| *Tbx5* | F: AGAACACTGCGTTTTGCACC |
|  | R: TGCATCCGAGACATCCTGTG |
| *Isl1* | F: CCGGAGAGACATGATGGTGG |
|  | R: GCAGGCTGATCTATGTCGCT |
| *Smarcd3* | F: AACGCAGGGCTGAGTTCTAC |
|  | R: CTGCGCTGCTGGATCTTACA |
|  |  |

**Table S3. Usage information of antibodies**

| Name | Application | Vendor | Product No. |
| --- | --- | --- | --- |
| Nkx2.5 | Westen blotting | Santa Cruz Biotechnology | Sc-376565 |
| Gata4 | Westen blotting | Cell Signaling Technologies | 36966S |
| Tbx5 | Westen blotting | Santa Cruz Biotechnology | Sc-376952 |
| Isl1 | Westen blotting | Developmental Studies Hybridoma Bank | 39.3F7 |
| Smarcd3  (Baf60c) | Westen blotting | Santa Cruz Biotechnology | Sc-101163 |
| β-actin | Westen blotting | Cell Signaling Technologies | 4970S |
| Cardiac α-actinin | Immunostaining | Sigma-Aldrich | A7811 |
| Cardiac α-actinin (ACTN2) | Immunostaining | Proteintech | 14221-1-AP |
| cTnT | Immunostaining | Proteintech | 15513-1-AP |
| αSMA | Immunostaining | Invitrogen | 53-9760-82 |
| SM-MHC (MYH11) | Immunostaining | Abcam | AB224804 |
| Anti-Rabbit HRP  Anti-Mouse HRP | Westen blotting  Westen blotting | Cell Signaling Technologies  Cell Signaling Technologies | 7074S  7076S |
| Anti-Mouse Alexa Fluor 488  Anti-Rabbit Alexa Fluor 488 | Immunostaining  Immunostaining | Invitrogen  Invitrogen | A28175  A11008 |
|  |  |  |  |
|  |  |  |  |
