# Supplementary material for: In vivo CRISPR-mediated activation of cardiogenic genes to reprogram cardiac fibroblasts
Source: Front Cell Dev Biol. 2026 Apr 22;14:1812941. doi: 10.3389/fcell.2026.1812941 (PMC13143979; doi:10.3389/fcell.2026.1812941)
Supplement: Supplementary file 2 [file DataSheet1.pdf]

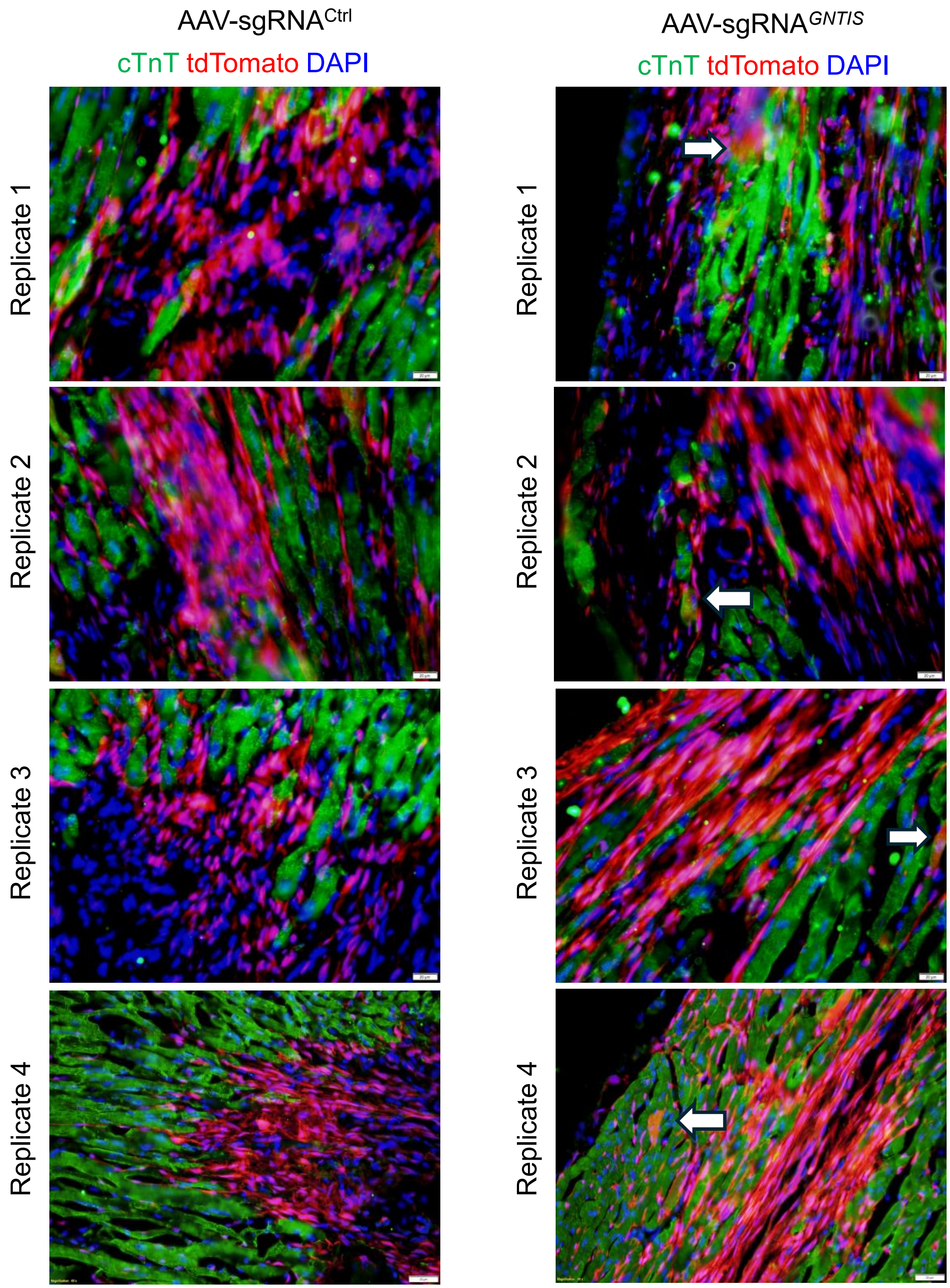

Figure S1. Lineage tracing of CF-derived cardiomyocytes. $cTnT^+$ cells derived from $tdTomato^+$ CFs (highlighted by white arrow) were examined 8 weeks post-MI. Representative images from each experimental group are shown, and biological replicates (individual animals) were used to assess reprogramming efficiency.

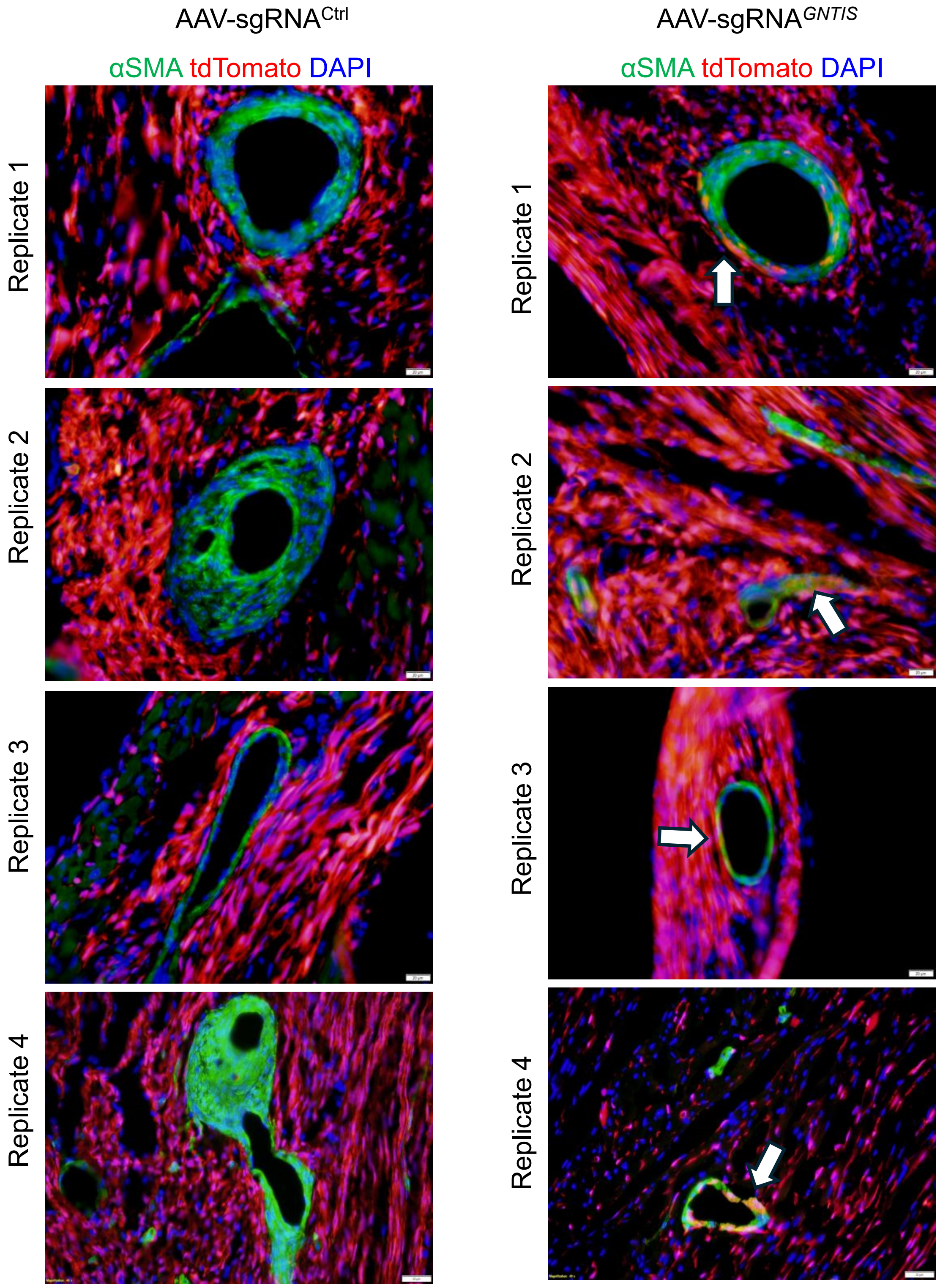

Figure S2. Lineage tracing of CF-derived smooth muscle cells. αSMA$^+$ cells derived from tdTomato$^+$ CFs (highlighted by white arrow) were examined 8 weeks post-MI. Representative images from each experimental group are shown, and biological replicates (individual animals) were used to assess reprogramming efficiency.
